# Prevalence and Types of Drugs Used Among Hepatitis A Patients During Outbreaks Associated with Person-to-Person Transmission, Kentucky, Michigan, and West Virginia, 2016–2019

**DOI:** 10.13023/jah.0401.06

**Published:** 2022-02-13

**Authors:** Megan G. Hofmeister, Alice Asher, Christopher M. Jones, Ryan J. Augustine, Cole Burkholder, Jim Collins, Monique A. Foster, Shannon McBee, Douglas Thoroughman, Erica D. Thomasson, Mark K. Weng, Phillip R. Spradling

**Affiliations:** 1 Centers for Disease Control and Prevention; 2 Michigan Department of Health and Human Services; 3 West Virginia Department of Health and Human Resources; 4 Kentucky Department for Public Health

**Keywords:** Appalachia, hepatitis A, methamphetamine, opioids, disease outbreaks, United States

## Abstract

**Background:**

People who use drugs are at increased risk for hepatitis A virus infection. Since 1996, the Advisory Committee on Immunization Practices has recommended hepatitis A vaccination for people who use drugs. Since 2016, the U.S. has experienced widespread hepatitis A outbreaks associated with person-to-person transmission.

**Purpose:**

To describe the prevalence of drug use, route of use, and drugs used among hepatitis A outbreak-associated patients.

**Methods:**

State outbreak and medical records were reviewed to describe the prevalence, type, and route of drug use among a random sample of 812 adult outbreak-associated hepatitis A patients from Kentucky, Michigan, and West Virginia during 2016–2019. Differences in drug-use status were analyzed by demographic and risk-factor characteristics using the *X*^2^ test.

**Results:**

Among all patients, residents of Kentucky (55.6%), Michigan (51.1%), and West Virginia (60.1%) reported any drug use, respectively. Among patients that reported any drug use, methamphetamine was the most frequently reported drug used in Kentucky (42.3%) and West Virginia (42.1%); however, opioids were the most frequently reported drug used in Michigan (46.8%). Hepatitis A patients with documented drug use were more likely (*p*<0.05) to be experiencing homelessness/unstable housing, have been currently or recently incarcerated, and be aged 18–39 years compared to those patients without documented drug use.

**Implications:**

Drug use was prevalent among person-to-person hepatitis A outbreak-associated patients, and more likely among younger patients and patients experiencing homelessness or incarceration. Increased hepatitis A vaccination coverage is critical to prevent similar outbreaks in the future.

## INTRODUCTION

Hepatitis A is a vaccine-preventable disease of the liver, typically acquired through fecal–oral transmission of the hepatitis A virus (HAV) from direct person-to-person contact or consumption of contaminated food or water. Hepatitis A outbreaks among people who use drugs occurred in multiple states during the 1980s,^1^,^2^ contributing to the 1996 recommendation by the Advisory Committee on Immunization Practices (ACIP) that people who use drugs, both by injection and non-injection routes, be vaccinated against hepatitis A because of their increased risk for HAV infection.^3^

Since 2016, the U.S. has experienced hepatitis A outbreaks associated with person-to-person transmission that are unprecedented in the vaccine era. As of February 4, 2022, state health departments have reported more than 43,600 outbreak-associated patients across 37 states.^4^ Although the infections have spread primarily through close contact among people who use drugs,^4^ little is known about the characteristics of drug use that might be contributing to these outbreaks. The purpose of this analysis was to describe the prevalence of drug use, route of use, and drugs used among hepatitis A outbreak-associated patients (hereafter referred to as hepatitis A patients) in three heavily affected states.

## METHODS

A retrospective observational study of hepatitis A patients with onset between July 1, 2016, and June 10, 2019, was previously conducted.^5^ Study-eligible patients were residents of Kentucky, Michigan, and West Virginia and had been designated by their respective state health department as an outbreak-associated hepatitis A patient. State outbreak and hospital records among a random sample of 10% of hepatitis A patients from each of the three states were reviewed.

For this analysis, conducted using the previously collected dataset, data on drug type and route of use during a patient’s exposure period (i.e., the 15–50 days before symptom onset) were obtained through a combination of self-report during health department case investigations and urine drug test results from medical records associated with the clinical encounter when hepatitis A was diagnosed. Drug-type categories assessed included benzodiazepines, cannabis (regardless of state legalization status), cocaine, methamphetamine, and opioids. The analysis was restricted to illicit use of drugs by cross-referencing prescription lists in medical records to ensure that patients with documented benzodiazepine or opioid use did not have prescriptions for those drugs.

Descriptive statistics were calculated detailing the prevalence of drug use among hepatitis A patients in the three study states. Differences in drug use status by demographic (gender and age) and risk factor (homelessness or unstable housing and incarceration) characteristics were analyzed using a chi-square (X^2^) test. Age was dichotomized according to the mean age of the analytic sample into 18–39 years and ≥40 years. Risk factors were assessed during a patient’s exposure period. All analyses were performed using SAS, version 9.4 (SAS Institute Inc., Cary NC). Data collection was performed in the context of outbreak investigation and control. CDC determined this analysis did not constitute human subjects research; IRB review was not required.

## RESULTS

Eight hundred twelve hepatitis A patients aged ≥18 years were identified in the random samples from the three study states: 467 from Kentucky, 92 from Michigan, and 253 from West Virginia. Overall, patients were primarily male (62.7%, 509/812) with a mean age of 39.2 years; 11.3% (92/812) reported homelessness or unstable housing, and 11.6% (94/812) were currently or recently incarcerated at the time of hepatitis A diagnosis. Among all patients, 25.2% (205/812) had documentation of a urine drug test (22.7% of Kentucky residents, 19.6% of Michigan residents, and 32.0% of West Virginia residents).

Among patients, 55.7% (260/467) of Kentucky residents, 51.1% (47/92) of Michigan residents, and 60.1% (152/253) of West Virginia residents reported any drug use (Table 1). Among those with known drug-use status, injection was more common as the route of use than non-injection in Kentucky (57.6% vs. 35.4%), while non-injection was more common than injection in Michigan (45.2% vs. 32.1%); both routes of use were similarly common in West Virginia (54.5% vs. 53.6%) (Table 1). At least one-fifth of patients with known drug-use status reported both injection and non-injection drug use (23.8% Kentucky, 21.4% Michigan, and 36.5% West Virginia).

**Figure 1:**
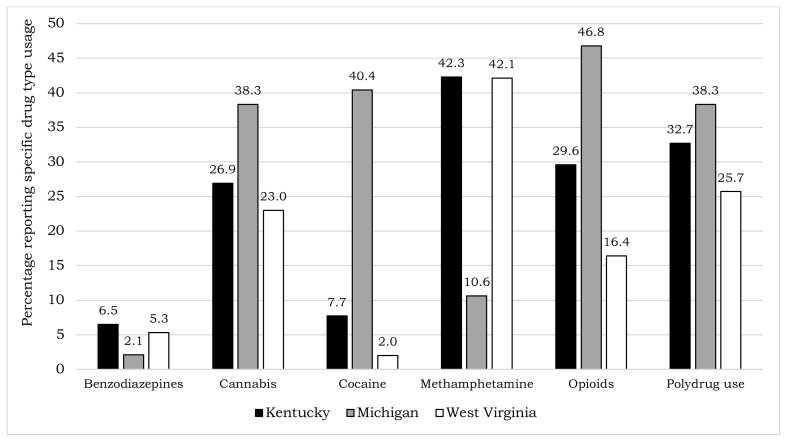
Proportion of specific drug types used among hepatitis A outbreak-associated patients who reported any drug use—Kentucky (n=260), Michigan (n=47), and West Virginia (n=152), 2016–2019. Drug-type categories are not mutually exclusive.

Among those who reported any drug use, methamphetamine was the most frequently reported type of drug used in Kentucky (42.3%) and West Virginia (42.1%); however, opioids were most frequently reported in Michigan (46.8%) (Figure 1). Benzodiazepines were the drug type least frequently reported by patients who reported any drug use in Kentucky (6.5%) and Michigan (2.1%), while cocaine was the drug type least frequently reported in West Virginia (2.0%) (Figure 1).

Hepatitis A patients with documented drug use were more likely to be experiencing homelessness or unstable housing than those without documented drug use (81.5% versus 18.5%, p<0.001); to have been incarcerated at the time of hepatitis A diagnosis or during their exposure period than those without documented drug use (67.0% versus 33.0%, p=0.03); and to be aged 18–39 years versus ≥40 years (64.1% versus 35.9%, p<0.001) than those patients without documented drug use. Drug use did not significantly differ by gender (55.8% of females versus 57.0% of males, p=0.74).

## IMPLICATIONS

Drug use has been recognized as a risk factor for HAV infection, and consequently has been an ACIP-recommended indication for hepatitis A vaccination for over 25 years. However, while significant attention has focused on the role of drug use in outbreaks of HIV and rising rates of hepatitis C virus infections in the U.S. in recent years, the contribution of drug use to the ongoing hepatitis A outbreaks associated with person-to-person transmission has largely been unexplored. This study provides the most comprehensive information available to date regarding the epidemiology of drug use in the ongoing U.S. hepatitis A outbreaks. It found that more than half of hepatitis A patients reported drug use, that injection and non-injection routes of use were both prevalent, and that the frequency of specific drugs used varied geographically. Additionally, hepatitis A patients with reported drug use were more likely to be experiencing homelessness or unstable housing, be currently or recently incarcerated, and be aged 18–39 years versus ≥40 years.

The prevalence of drug use found in this study is substantially higher than that of the general U.S. population. According to the 2019 National Survey on Drug Use and Health (NSDUH), 20.8% of noninstitutionalized US civilians aged 12 years or older reported past-year drug use compared with the >50% of hepatitis A patients reporting drug use in this analysis.^6^ This finding underscores the association of drug use with HAV infection, and the critical need to provide substance-use treatment and harm-reduction services as an integral part of the ongoing response to the U.S. hepatitis A outbreaks.

Of particular concern, the percentage of hepatitis A patients reporting methamphetamine use in the three states examined was extraordinarily high compared to the overall prevalence of methamphetamine use among adults in these states reported in NSDUH in 2018–2019: 42.3% versus 1.3% in Kentucky, 10.6% versus 0.4% in Michigan, and 42.1% versus 1.5% in West Virginia.^7^ These high rates of methamphetamine use among hepatitis A patients are consistent with other studies indicating a resurgence of methamphetamine-related harms in the U.S. For example, Jones et al. (2020) recently reported that methamphetamine-related substance-use treatment admissions in the U.S. increased from 15.1% of drug-related treatment admissions in 2008 to 23.6% in 2017.^8^ Methamphetamine use has been identified as a contributor to recent HIV outbreaks, rising hepatitis C virus infections, and other resurgent infectious diseases.^9^–^11^ Future research should further explore the link between methamphetamine use and HAV infections identified in this analysis.

This report is subject to limitations. Drug-use data for approximately 75% of patients in the random sample were self-reported and subject to recall and social desirability biases. Furthermore, information on drug use was missing for approximately 25% of patients. Consequently, the results of the analysis likely underestimate the actual overall prevalence of drug use among the outbreak-associated patients. Additionally, only three states experiencing hepatitis A outbreaks associated with person-to-person transmission were included in this analysis, and findings from other states experiencing outbreaks might differ because drug use varies geographically across the U.S. Although the analysis was conducted on random samples of hepatitis A patients from each of the three states, it is possible that particular areas within states were potentially overrepresented given that the outbreak burden varied geographically within each state.

The majority of patients in this analysis reported drug use. Drug use is one of the longest-standing ACIP-recommended indications for hepatitis A vaccination in the U.S. Many of the infections detailed in our study could have been prevented if existing public health recommendations and clinical guidance had been more effectively implemented in the years since ACIP first issued its recommendation. Unfortunately, this phenomenon is not restricted to the states included in this analysis. Nationally, many people at-risk for HAV infection due to drug use do not appear to be protected: a recent National Health and Nutrition Examination Survey study found that only 26.9% of U.S.-born adults aged ≥20 years during 2007–2016 who reported injection drug use reported ever being vaccinated against hepatitis A.^12^ Increased hepatitis A vaccination coverage, through improved universal and catch-up childhood hepatitis A vaccination, along with implementation of novel vaccination strategies by healthcare providers, substance-use treatment programs, and service providers for people who use drugs, will be necessary to help control the ongoing outbreaks and prevent similar outbreaks in the future.

**SUMMARY BOX**

**What is already known about this topic?**

People who use drugs are at increased risk for hepatitis A virus infection and have been recommended for hepatitis A vaccination by the Advisory Committee on Immunization Practices since 1996. Since 2016, the U.S. has experienced hepatitis A outbreaks associated with person-to-person transmission that are unprecedented in the hepatitis A vaccine era; infections have spread primarily through close contact among people who use drugs.

**What is added by this report?**

This report provides the most comprehensive information available to date regarding the epidemiology of drug use in the ongoing U.S. hepatitis A outbreaks associated with person-to-person transmission. Among patients reporting any drug use, methamphetamine was the most frequently reported drug used in Kentucky (42.3%) and West Virginia (42.1%); however, opioids were the most frequently reported drug class used in Michigan (46.8%).

**What are the implications for future research?**

Future research should further explore the link between methamphetamine use and HAV infections identified in this analysis.

## Figures and Tables

**Table 1:** Prevalence of drug use and route of drug use among hepatitis A outbreak-associated patients—Kentucky, Michigan, and West Virginia, 2016–2019

	Proportion of hepatitis A outbreak-associated patients ≥18 years old reporting drug use		
	Kentucky (n=467), n (%)	Michigan (n=92), n (%)	West Virginia (n=253), n (%)
Any drug use			
Yes	260 (55.7)	47 (51.1)	152 (60.1)
No	68 (14.6)	37 (40.2)	59 (23.3)
Missing	139 (29.8)	8 (8.7)	42 (16.6)
Route of use*			
Injection drug use			
Yes	189 (57.6)	27 (32.1)	113 (53.6)
No	82 (25.0)	54 (64.3)	88 (41.7)
Missing	57 (17.4)	3 (3.6)	10 (4.7)
Non-injection drug use			
Yes	116 (35.4)	38 (45.2)	115 (54.5)
No	68 (20.7)	40 (47.6)	70 (33.2)
Missing	144 (43.9)	6 (7.1)	26 (12.3)

* Restricted to those with known drug use status (n=328 for Kentucky, n=84 for Michigan, and n=211 for West Virginia). Route-of-use categories are not mutually exclusive.
